# Supplementary material for: Genome-Wide Identification, Characterization and Expression Analysis of the JAZ Gene Family in Resistance to Gray Leaf Spots in Tomato
Source: Int J Mol Sci. 2021 Sep 15;22(18):9974. doi: 10.3390/ijms22189974 (PMC8469637; doi:10.3390/ijms22189974)
Supplement: Supplementary file 1 [file ijms-22-09974-s001.zip › ijms-1340821-sup/Supplementary materials/Supplementary Table S1.pdf]

Supplementary Table S1: Gene descriptions and protein characteristics of 26 members of JAZ gene family in tomato.

Gene name	Gene ID	CDS length (bp)	Chr	Position（5'-3'）	Protein length (aa)	Protein characteristics					Subcellular location
						MW (kDa)	Theoretical pI	Instability Index	Aliphatic Index	GRAVY	
SlJAZ1	*Solyc01g005440.3*	1017	1	309090-312465	338	35.79	9.01	50.32	69.56	-0.259	Nucl
SlJAZ2	*Solyc01g009730.2*	375	1	3966238-3982582	124	14.01	4.99	38.56	74.68	-0.405	Cyto
SlJAZ3	*Solyc01g009740.2*	1125	1	4032844-4038809	374	42.12	8.93	47.22	83.13	-0.31	Nucl
SlJAZ4	*Solyc01g011097.1*	321	1	7924734-7925456	106	11.89	9.58	49.38	100.28	-0.065	Nucl
SlJAZ5	*Solyc01g011098.1*	444	1	7927099-7928483	147	16.49	9.76	20.36	106.05	-0.189	Nucl
SlJAZ6	*Solyc01g011175.1*	186	1	8009901-8010230	61	6.96	6.09	48.22	98.85	0.054	Chlo
SlJAZ7	*Solyc01g103600.3*	414	1	92055858-92057073	137	15.77	7.06	69.46	72.63	-0.836	Nucl
SlJAZ8	*Solyc01g106030.3*	999	1	93901607-93908706	332	36.01	8.47	42.67	60.54	-0.697	Nucl
SlJAZ9	*Solyc01g106040.3*	1137	1	93909674-93924139	378	41.2	5.08	55.37	61.9	-0.566	Nucl
SlJAZ10	*Solyc03g118540.3*	1173	3	68914104-68917741	390	41.08	9.33	55.2	78.74	-0.149	Cyto
SlJAZ11	*Solyc03g122190.3*	930	3	71585569-71589220	309	33.67	8.46	63.64	71.1	-0.646	Nucl
SlJAZ12	*Solyc04g076525.1*	738	4	61500578-61506539	245	27.58	9.35	31.31	66.82	-0.564	Nucl
SlJAZ13	*Solyc04g076527.1*	987	4	61506632-61516647	328	36.1	6.32	37.01	76.37	-0.356	Nucl

Gene name	Gene ID	CDS length (bp)	Chr	Position（5'-3'）	Protein length (aa)	Protein characteristics					Subcellular location
						MW (kDa)	Theoretical pI	Instability Index	Aliphatic Index	GRAVY	
SlJAZ14	*Solyc06g065650.3*	1284	6	41170308-41177948	427	44.86	9.04	49.57	63.09	-0.556	Nucl
SlJAZ15	*Solyc06g068930.2*	741	6	42875169-42876719	246	27.1	9.79	48.87	68.62	-0.697	Nucl
SlJAZ16	*Solyc06g084120.3*	1164	6	49388525-49395042	387	42.96	8.68	50.33	67.8	-0.715	Nucl
SlJAZ17	*Solyc07g042170.3*	651	7	55305593-55306887	216	23.85	8.53	44.43	69.63	-0.523	Cyto
SlJAZ18	*Solyc08g036660.3*	420	8	10494205-10495853	139	15.98	9.42	81.06	67.34	-0.781	Nucl
SlJAZ19	*Solyc08g036640.3*	339	8	10547251-10548057	112	12.82	9.74	93.54	67.05	-0.762	Nucl
SlJAZ20	*Solyc08g036620.3*	312	8	10664074-11204533	103	12.1	9.94	75.83	84.27	-0.777	Chlo
SlJAZ21	*Solyc08g036505.1*	342	8	11183576-11204533	113	12.8	5.25	55.24	83.63	-0.529	Nucl
SlJAZ22	*Solyc09g065630.3*	1020	9	64028285-64034795	339	37.56	7.09	55.42	68.85	-0.711	Nucl
SlJAZ23	*Solyc10g047640.2*	975	10	41464370-41477236	324	34.44	5.61	43.1	61.14	-0.622	Nucl
SlJAZ24	*Solyc11g011030.2*	687	11	4085805-4089166	228	26.37	7.86	49.05	66.36	-0.756	Nucl
SlJAZ25	*Solyc12g009220.2*	759	12	2502493-2504654	252	28.16	9.3	46.27	56.51	-0.795	Nucl
SlJAZ26	*Solyc12g049400.2*	603	12	61897549-61902508	200	22.36	9.13	40.84	71.3	-0.651	Nucl

Abbreviations: MW, Molecular weight; GRAVY, Grand average of hydropathicity; Nucl, nucleus; Cyto, cytoplasm; Chlo, chloroplast.
