# Supplementary figures and images for: Genome-Wide Identification, Characterization and Expression Analysis of the JAZ Gene Family in Resistance to Gray Leaf Spots in Tomato
Source: Int J Mol Sci. 2021 Sep 15;22(18):9974. doi: 10.3390/ijms22189974 (PMC8469637; doi:10.3390/ijms22189974)

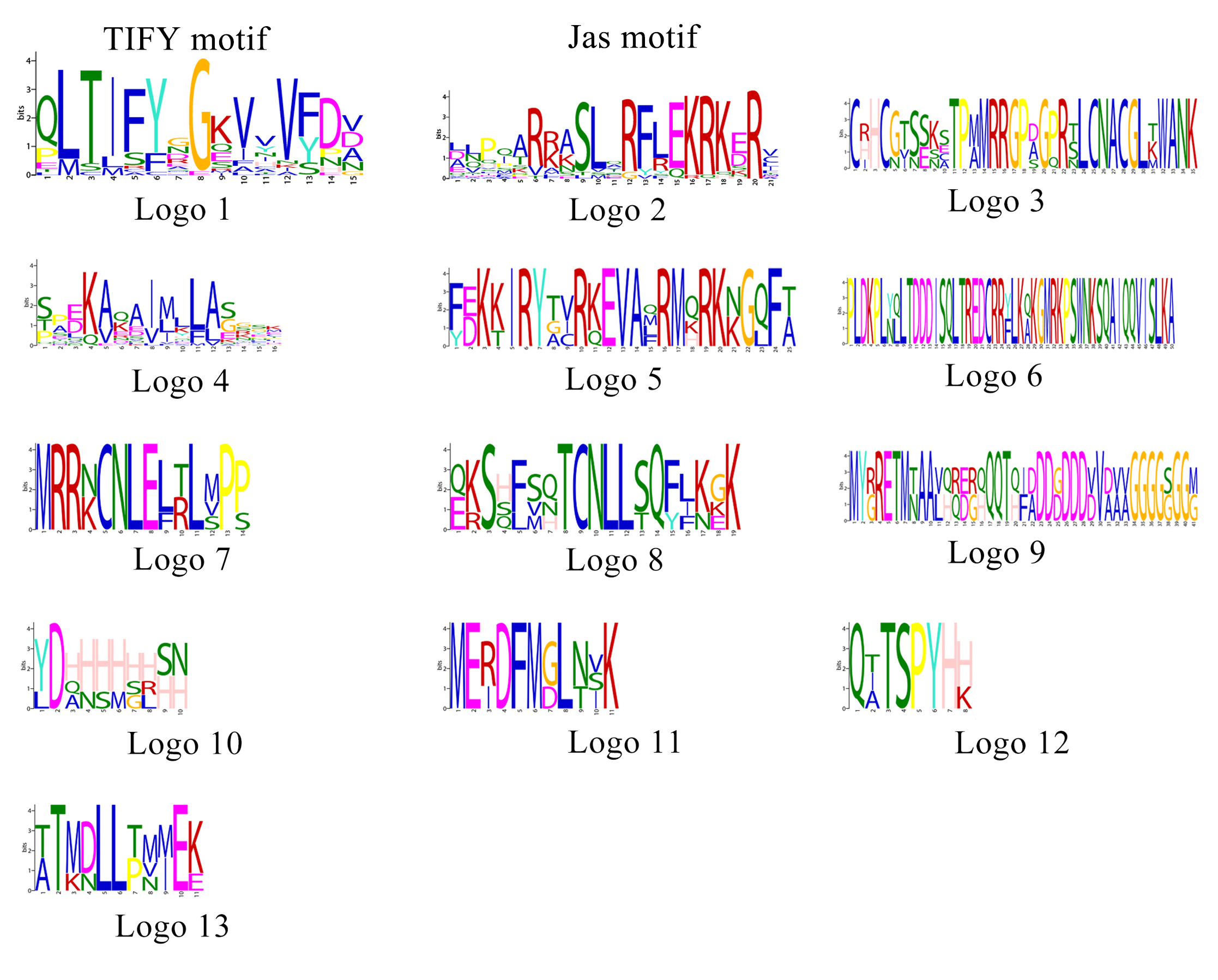

Supplement: Supplementary file 1 [file ijms-22-09974-s001.zip › ijms-1340821-sup/Supplementary materials/Supplementary Figure S1.jpg]

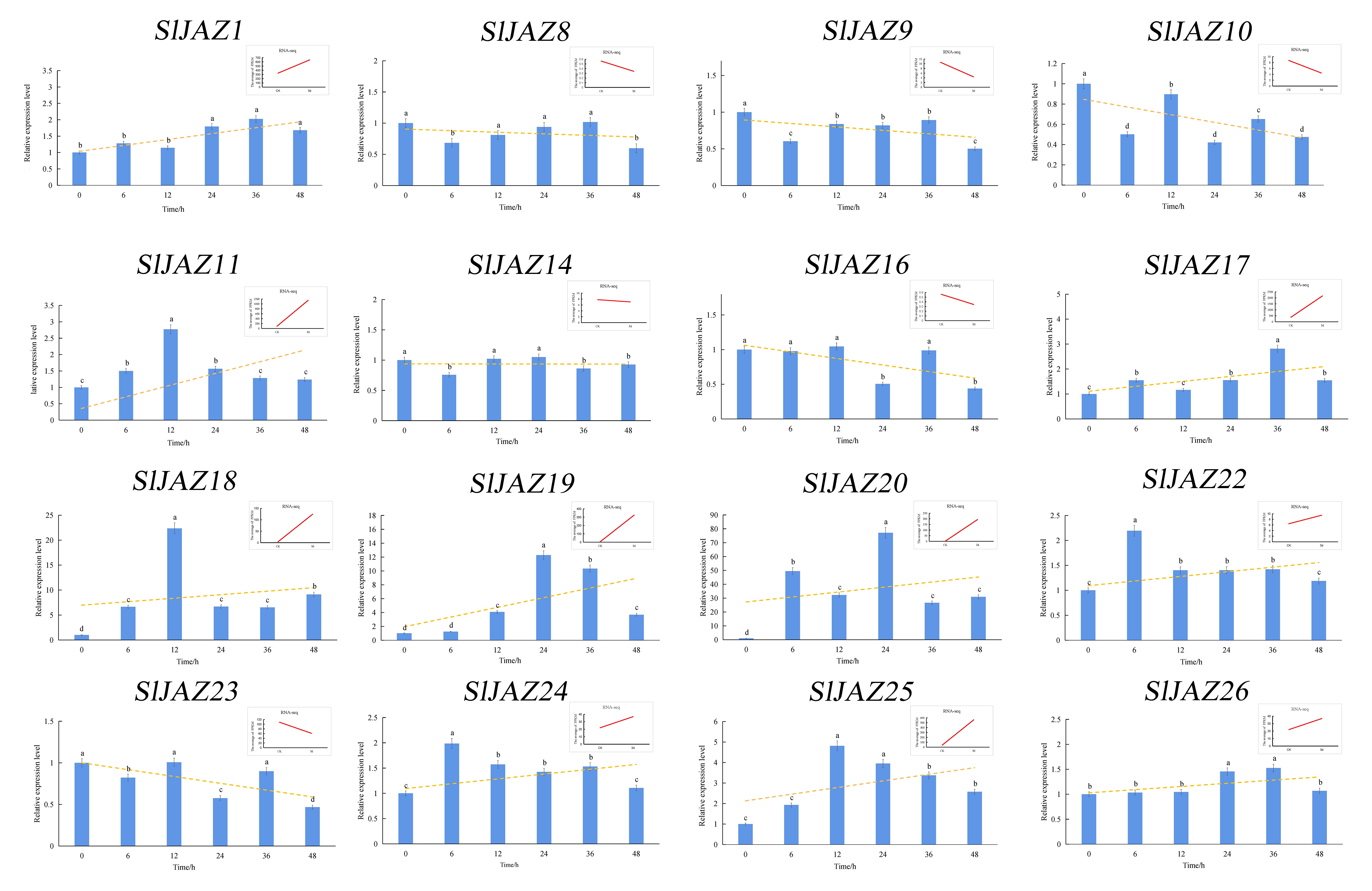

Supplement: Supplementary file 1 [file ijms-22-09974-s001.zip › ijms-1340821-sup/Supplementary materials/Supplementary Figure S2.jpg]

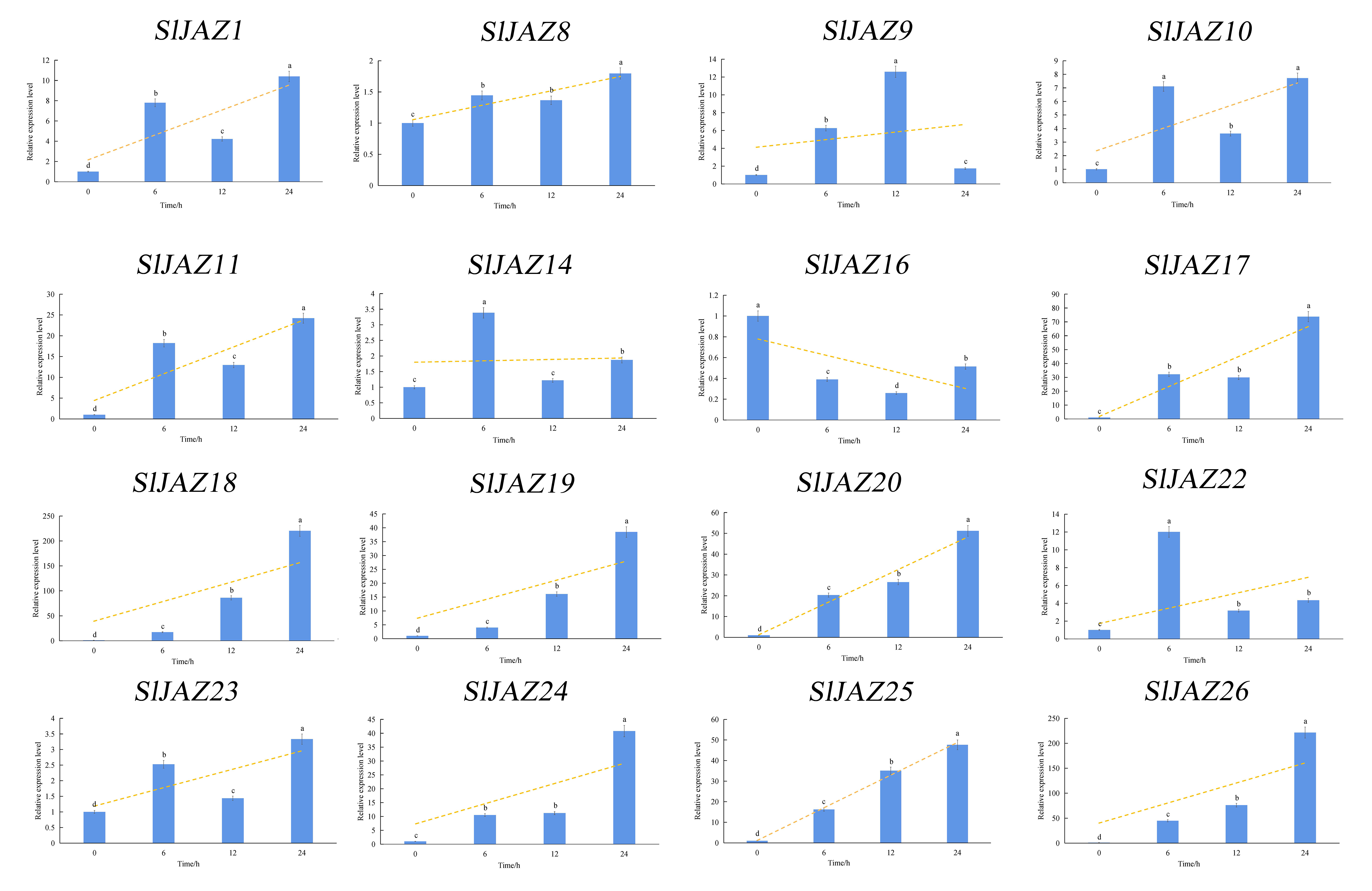

Supplement: Supplementary file 1 [file ijms-22-09974-s001.zip › ijms-1340821-sup/Supplementary materials/Supplementary Figure S3.jpg]

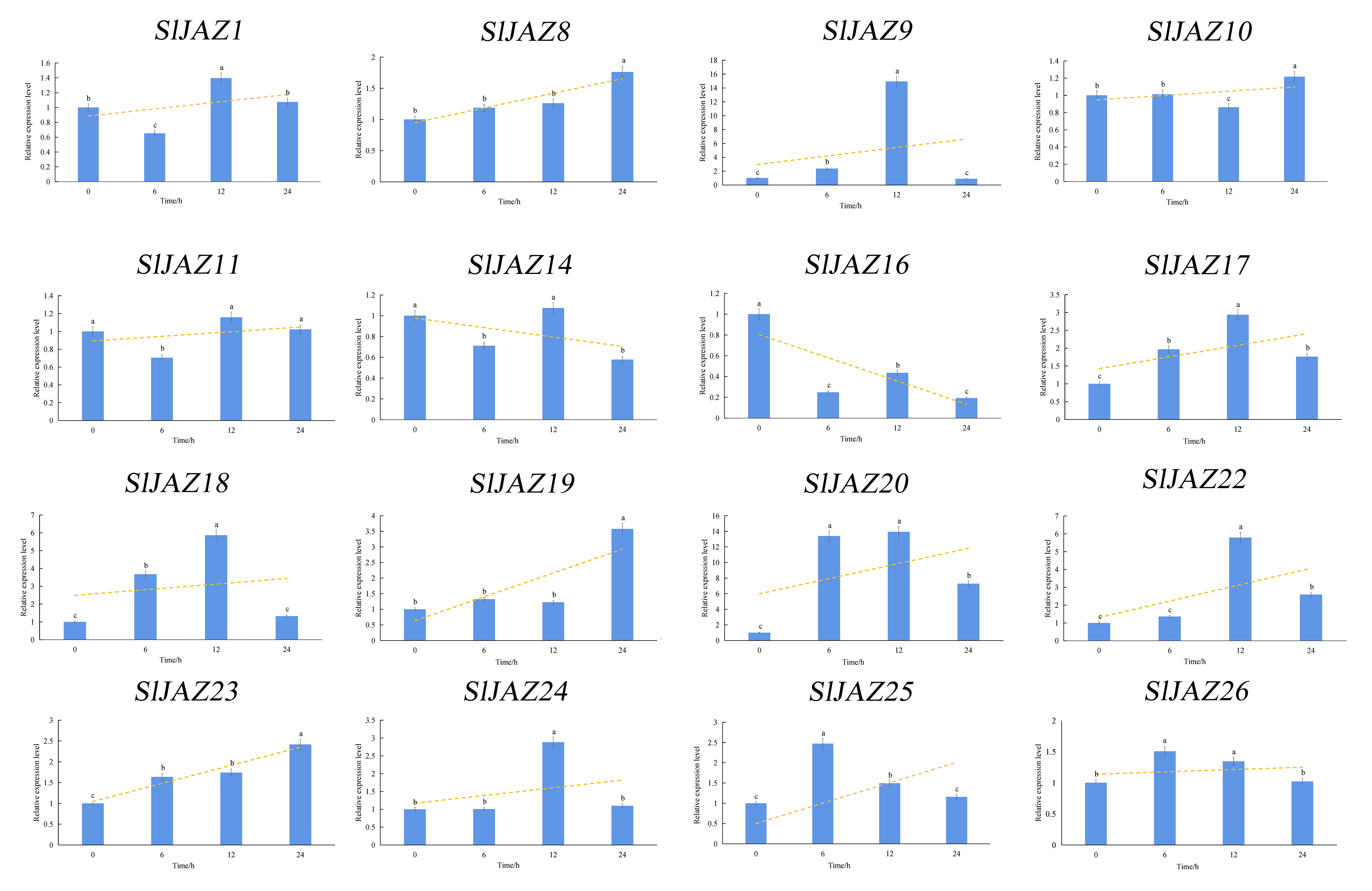

Supplement: Supplementary file 1 [file ijms-22-09974-s001.zip › ijms-1340821-sup/Supplementary materials/Supplementary Figure S4.jpg]

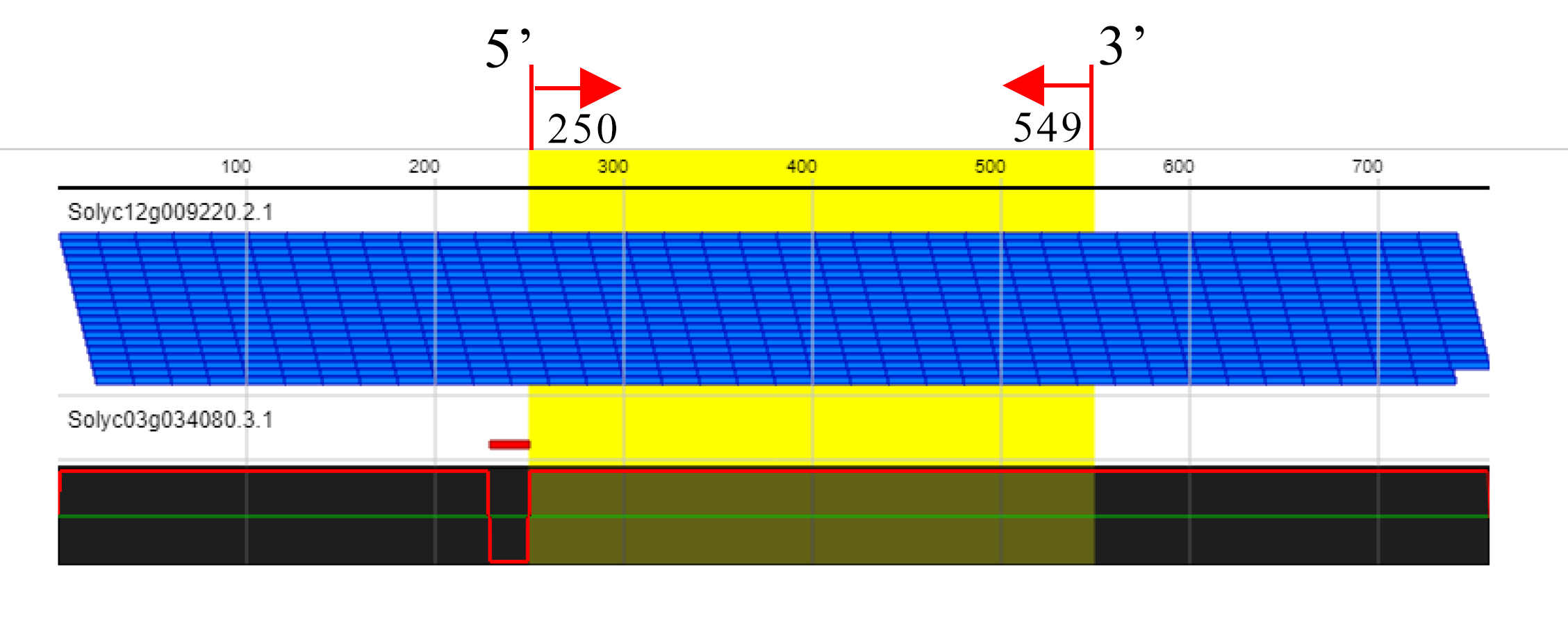

Supplement: Supplementary file 1 [file ijms-22-09974-s001.zip › ijms-1340821-sup/Supplementary materials/Supplementary Figure S5.jpg]
